# *In vitro *hydrodynamic properties of the Miethke proGAV hydrocephalus shunt

**DOI:** 10.1186/1743-8454-3-9

**Published:** 2006-06-29

**Authors:** David M Allin, Zofia H Czosnyka, Marek Czosnyka, Hugh K Richards, John D Pickard

**Affiliations:** 1 Academic Neurosurgical Unit, Addenbrooke's Hospital, Cambridge CB2 2QQ, UK

## Abstract

**Background:**

Adjustable shunts are very popular in the management of hydrocephalus and are believed to help in minimizing the number of surgical revisions. The drawback with almost all constructions is that they may be accidentally readjusted in relatively weak magnetic fields (around 30–40 mTesla)

**Materials and methods:**

The ProGav Miethke shunt is composed of an adjustable ballon-spring valve unit and an integrated over-drainage compensating gravitational device (known as the shunt assistant). A mechanical 'brake' is intended to prevent changes to the valve's performance level in a strong magnetic field. We evaluated the performance and hydrodynamic properties of a sample of three valves in the UK Shunt Evaluation Laboratory.

**Results:**

All the shunts showed good mechanical durability over the three-month period of testing, and good stability of hydrodynamic performance over a one-month period

The pressure-flow performance curves, operating, opening and closing pressures fell within the limits specified by the manufacturer, and changed according to the programmed performance levels. The operating pressure increased when the shunt assistant was in the vertical position, as specified. The valve has a low hydrodynamic resistance (0.53 mm mmHg ml^-1 ^min^-1^). External programming proved to be easy and reliable. Strong magnetic fields from a 3 Tesla MR scanner were not able to change the programming of the valve.

**Conclusion:**

The ProGAV shunt is an adjustable, low resistance valve that is able to limit posture-related over-drainage. Unlike other adjustable valves, the ProGAV cannot be accidentally re-adjusted by external magnetic field such as a 3T MR scanner.

## Background

A hydrocephalus shunt drains excess cerebrospinal fluid **(CSF) **to elsewhere in the body. Ideally, the shunt should restore a normal CSF circulation. Therefore, the rate of drainage should be proportional to the positive difference between the CSF pressure and the sagittal sinus pressure. With a negative pressure difference, the drainage should cease. Full restoration of this mechanism is unrealistic because most shunts drain fluid into the peritoneal or atrial cavities not into the sagittal sinus. Hence, the driving pressure is a pressure difference between inlet (ventricles) and outlet (peritoneal or atrial) compartments. The other requirements of an ideal shunt are:

• The resistance of an open shunt taken together with the natural CSF outflow resistance (usually increased in hydrocephalus) should be close to the normal resistance to CSF outflow, i.e. 6 to 10 mmHg ml^-1 ^min^-1 ^[1]

• Flow should remain constant under constant pressure conditions.

• Flow through the shunt should not depend on the body posture or be affected by body temperature, external (environmental) pressure (within the physiological range for subcutaneous pressure) or the pulsatile component of CSF pressure.

• Opening and closing pressures (the pressure at which flow starts and ceases) should remain constant under the conditions listed above.

• Reversal of flow through the shunt should be impossible.

When programmable shunts became available they for the first time allowed fine-tuning of their operating pressure *in vivo*. This enabled any mismatch between the shunt's operating pressure and the requirements for 'optimal' ICP, i.e. the pressure that gives the best and quickest relief from symptoms, to be adjusted without the need for surgery to replace the shunt. However, it was found that following MRI scanning, a diagnostic procedure frequently used on patients with hydrocephalus, almost all programmable shunts had their settings changed by the powerful magnets used by the scanning equipment [2]. Further studies have been published, indicating that practically all models of programmable valves can be accidentally readjusted by magnetic fields as low as a fraction of 10^-2 ^Tesla [3-5]. This increases the risk of accidental re-adjustment of the valve by household items such as magnetic toys [4], holders and furniture latches.

The newly released Miethke proGAV^® ^shunt is a programmable valve with an integrated siphon compensating gravitational device [6]. It incorporates a brake system to prevent the shunt being reset by an external magnetic field of up to 3 Tesla [7].

The aim of this study was to assess the hydrodynamic properties of the shunt and the efficacy of its design features in order to provide accurate and independent information for the neurosurgeon selecting a shunt.

## Materials and methods

### Shunt

The ProGAV shunt (Miethke GMBH & Co KG, Potsdam, Germany) is composed of two units: an adjustable unit and a non-adjustable gravitational shunt assistant. Both the units have titanium shells.

**Figure 1:**
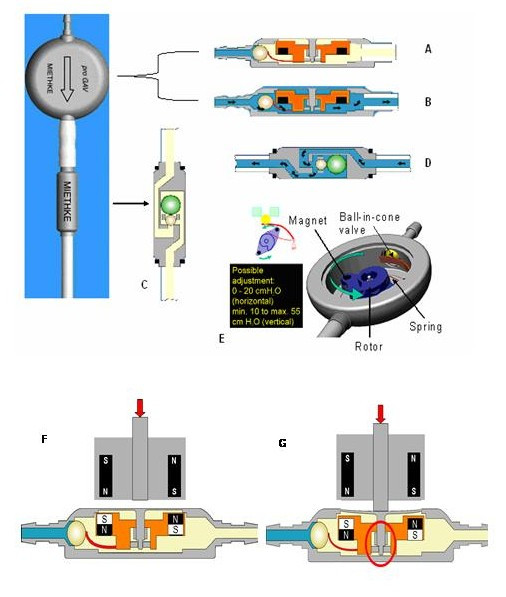
Diagrams of the Miethke proGAV^® ^shunt adapted from  with the permission of authors. A: Adjustable unit in 'closed' state. The ball-in-cone valve is closed and drainage is blocked. B: Adjustable unit in 'open' state. Differential pressure overcomes the spring force. The ball moves out of the cone and the gap opens, allowing drainage. C: Gravitational unit in vertical position. When patient is upright unit closes, increasing effective opening pressure of the valve. Drainage occurs when the differential pressure exceeds the combined opening pressures of both unit. D: Gravitational unit in horizontal position. The unit is open and the opening pressure of the valve is determined only by the adjustable unit. E: Internal adjustment mechanism of the shunt with details of profiled rotor controlling pre-load of the spring supporting ball F: The magnetic tool is used to turn the rotor. In neutral position rotor cannot move even in a presence of very strong magnetic field (up to 3T) as the brake is engaged. G: The turning is only possible when the central part of valve's casing is depressed, releasing the brake.

The adjustable unit uses a ball-in-cone valve system. The tension of the spring holding the ball in place can be adjusted by turning the rotor (torsion bar) using the external magnetic adjustment tool, thus changing the operating pressure. The valve has a brake system that holds the rotor in place to prevent unwanted re-adjustment when the shunt is exposed to an external magnetic field. To release the brake, a downward force (800 to 1600 gram-force) is applied to the unit using the adjustment tool (Figure 1). The valve has a diameter of 18 mm. It has a relatively large internal volume compared to other models, which is intended to minimize the risk of obstruction.

The gravitational unit (shunt assistant) increases the opening pressure of the shunt when the patient is vertical by blocking the inlet flow using a gravity-assisted ball bearing. If the patient is horizontal, the ball no longer blocks the opening and the unit provides substantially less resistance. When in the vertical position, the opening pressure of the ProGAV is the sum of the opening pressure of the adjustable unit and the gravitational unit. The gravitational unit is available with a graded cross-sectional area of aperture supporting the ball, which is intended to compensate for the 'siphoning' that occurs in CSF-filled tubing of different lengths. The setting of the shunt assistant, expressed in cm should be equivalent to the distance between the right atrium of the heart and the outlet of the peritoneal catheter.

The valve can be implanted on the chest rather than on the skull. Although the position of the valve is not important, the shunt assistant should be always parallel to the longitudinal body axis.

A special compass and an adjustment tool are supplied: The compass reads the valve's current setting and can also be used to pinpoint the shunt's location if it cannot be found by simple palpation. The adjustment instrument is used to change the setting of the shunt, allowing *transcutaneous *adjustment. To adjust the shunt, force must be applied onto its superior surface with the tool to unlock the braking mechanism. The adjustment tool's built in magnet then moves the internal mechanism of the shunt to set it at the desired resistance.

### Testing rig and protocol

The detailed description of testing rig and methodology has been published previously [7]. Three Miethke proGAV shunts were mounted in three identical testing rigs. The shunts were submerged in a water bath at a constant temperature of 37°C and at a depth of approximately 1 cm. De-ionised and de-aerated water, from either the syringe infusion pump or the fluid container, was used to perfuse the shunt. A pulse pressure generator was used to simulate the pulsation of ICP seen *in-vivo*. The input pressure to the shunt was measured using a luer lock pressure transducer that was placed at the same level as the shunt. Fluid flowing through the shunt was collected in a container placed on an electronic balance.

Measurement was controlled by a standard IBM compatible personal computer with software designed in-house. The flow rate through the shunt was calculated by the program, which read and zeroed the balance periodically. Weighing the fluid outflow in increments enabled the influence of fluid vaporisation from the outlet container to be cancelled. In this way the relationship between differential pressure and flow was evaluated using a pressure-flow performance curve. Opening and closing pressure, and hydrodynamic resistance of the opened shunt, were calculated.

Repeated tests were carried out over a three months period and included an assessment of how flow-pressure performance changed. Tests performed included: measurement over 28 consecutive days, at different settings, in different temperatures, in horizontal and vertical positions, with the presence of variable pulse amplitude, with and without distal drain, before and after a simulated scan in a 3 Tesla MRI magnet.

Linear regression was used to analyse the relation between measured closing pressure and valve pressure setting. Data are shown as mean values, with 95% confidence bars where appropriate. The significance for differences between different valves was tested using a t-test.

The study was conducted in the U.K. Shunt Evaluation Laboratory in Cambridge. Measurement protocol was a standard protocol used for assessment of hydrocephalus shunts [8]. The results were obtained and interpreted independently on the manufacturer.

## Results

**Figure 2:**
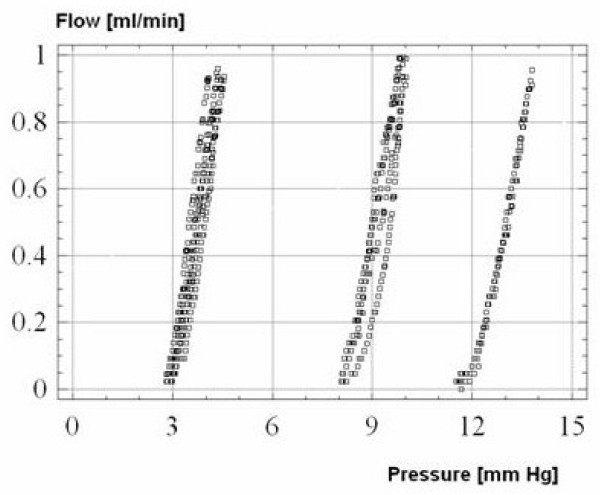
Shapes of pressure-flow curves for the three shunts tested at three different pressure settings: 4 cm H_2_O, 10 cm H_2_O and 16 cm H_2_O (left to right). The pressure units used for these measurements were mm Hg. However the manufacturer used cm H_2_0 for the settings on the shunts.

The typical pressure-flow curves for a valve, working at three different performance levels are presented in Figure 2. The valve, without a distal catheter and a pulsatile pressure wave, had almost linear characteristics.

The static resistance of the shunt was 0.53 mmHg ml^-1 ^min^-1 ^(95% confidence limit: +/- 0.04 mmHg ml^-1 ^min^-1^). The connection of a standard 76 cm distal catheter increased the resistance of the valve to around 3.0 mmHg ml^-1 ^min^-1^.

**Figure 3:**
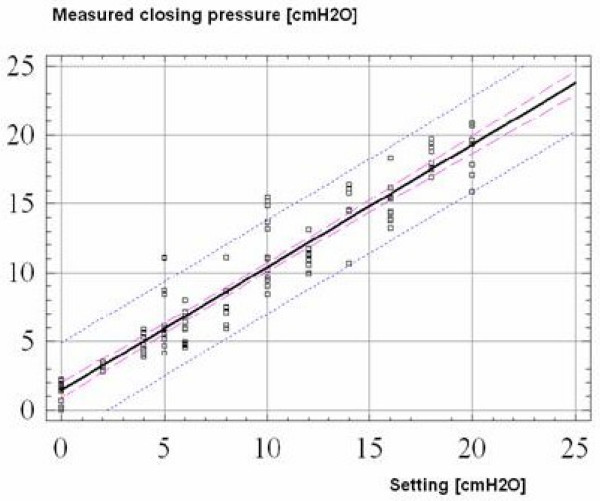
Linear regression plot between measurement points and settings of the valve (R = 0.97). The 95% confidence limits for predictors are represented by the outer dashed line and the means for each shunt by the inner dashed line.

Regression between measurement of the operating pressure and setting of the valves showed a significant linear relationship (R = 0.97) with a 95% limit for predictors +/- 3.5 mm Hg (Figure 3). The programming tool was very straightforward and easy to use. Reliability of programming was high, the valve was correctly adjusted in >95% cases. Verification of programming is quick and easy and it does not require an X-ray scan.

**Figure 4:**
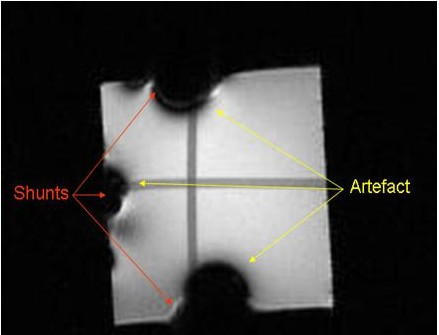
MRI scan of three shunts. The shunts were placed on testing block of glycerin (20 × 20 × 20 cm) and a 3T MRI scan was performed. Black areas show artifacts on MRI image.

All three shunts were scanned in a 3 Tesla MRI machine. We ran a routine T1/T2 weighted sequence and the timing of the MRI scanning was comparable to human scans (about 20 min). The result of the scan is shown in Figure 4, where the outlines of the shunts are visible as well as the large artefacts they produced. The shunts were then assessed to see if the setting or hydrodynamic properties had altered following the scan and no differences were found. Programming of the shunt was not affected by the high magnetic field exposure. If the artefacts are likely to pose a potential problem for brain scans, this could be avoided by placing the shunt over the chest.

When the shunt assistant was in the vertical position, the pressure-flow performance curve and operating pressure were shifted upwards. The amount of shift was equivalent to the change in the opening pressure of the shunt assistant due to its orientation, in this case 30 cmH_2_O. Within the tested range of 34° – 40°C, temperature had no significant effect on shunt performance. Pulse amplitude superimposed on inlet pressure had a tendency to decrease operating pressure by 3.5 mm Hg per 10 mm Hg of peak-to-peak amplitude.

No significant differences in measured parameters were found between the three valves tested. There was no significant difference detected in parameters over the 28-day test period. Preassembled junctions did not break when a test specimen was subjected to a load of 1 kg force for 1 min. All junctions remained free from leakage when the water pressure was increased to 3 kPa (about 25 mm Hg). The valve did not show any reflux when tested according to the International Standards Organisation (ISO) standard. Valves did not exhibit reversal of flow for an outlet-inlet differential pressure of up to 200 mm Hg.

## Discussion

Historically, unlike drugs, surgical devices have been introduced onto the market with a minimum of independent scrutiny, occasionally resulting in unfortunate incidents such as the problems associated with early models of silicone breast implants. There are also examples of shunts being withdrawn from the market when performance differed markedly from the manufacturer's specifications. However, more recently, ISO standards and randomized controlled trials have been established to assess the efficacy of surgical devices and help provide surgeons and their patients with safer products.

ISO 7197 (see ) describes a set of standards that ensures a minimum assessment of a valve's performance and examines the consistency of the manufacturing process that is thought to be adequate. Perhaps surprisingly, not all shunt manufacturers adhere to these standards [8]. As such, due to the varying and inconsistent data supplied by manufacturers concerning their product, it was considered important that formal studies, such as the one described here, were carried out by independent organisations. It was in this light that the UK shunt laboratory was created in 1993 [8].

Due to the limitations of this *in vitro *study it was not possible to assess whether the shunt conformed to all the ISO standards, but the results from the tests performed have been promising and meet the criteria required, in that the shunt performed to the manufacturer's specification. The programming of the shunt functioned as expected. Increasing the shunt's setting led to a similar increase in the observed operating pressure, as specified by the manufacturer. This trend was significant for all settings.

Good results were obtained with respect to testing the two key features of the ProGAV. The brake system performed satisfactorily, reducing the chance of accidental reprogramming of the shunt. The shunt was not affected by the magnets and is deemed to be MRI safe (*in vitro*) with respect to program resetting. However no system is infallible, and as the opening pressure setting can be very critical in some hydrocephalic patients it would perhaps be prudent for the shunt's setting to be checked after a scan.

The integrated anti-siphon device also performed well. A substantial increase in operating pressure was observed when the gravitational unit was orientated in a vertical position. Hopefully this should minimise the effect of any tendency to posture-related over-drainage.

The decrease in operating pressure seen when a pulse pressure was superimposed on a static differential pressure was noted, but this is common to all pressure-differential valves. [9]. Hence, flow through the valve may be affected by the pulsatile component of CSF pressure due to the cardiac pulse and breathing. As such it is important to remember that this is a laboratory study and that the hydrodynamic properties of the shunt may be different *in vivo*.

A very low hydrodynamic resistance was measured: 0.53 mmHg ml-1 min-1, much lower than the physiological value of 6 – 10 mmHg ml^-1 ^min [1]. Even with a standard peritoneal catheter (usually approximately 90 cm long and with an internal diameter of 1.2 mm) that has a resistance of 2.5–3.5 mmHg ml^-1 ^min-1 this would only increase the resistance to about 60% of the physiological value. As the catheter represents about 85% of overall resistance of the complete setup, care must be taken when shortening it.

This low resistance in the open state means that the valve can cope with a sudden build up of pressure and it might therefore be considered for use in the 20–30% of hydrocephalic patients experiencing intermittent intracranial hypertension [8]. Logically, for patients suffering from this type of hydrocephalus the choice of shunt is restricted to those with a low resistance. On the other hand, shunts with low resistance are often associated with an increased risk of over-drainage. If the over-drainage is posture related then the anti-siphoning device should reduce this problem. However if it is due to nocturnal vasogenic activity when the patient is horizontal then this will still remain an issue [9].

In the longer lasting pressure-flow tests, which last several hours, the shunt produced consistent patterns of flow that did not vary over the trial period. Hence the proGAV is expected to be a reliable shunt when used in patients.

One important question that remains is how well does the shunt's performance in the laboratory translate to its ability to restore CSF flow in the patient, given that the laboratory conditions mimic only in part conditions within the human body? This is a very complex question. There is very limited literature available on this subject and what there is appears to be contradictory. Performance is heavily dependent on a patient's individual CSF dynamics and therefore shunt implantation should ideally be done in conjunction with an analysis of each patient's dynamics [10].

A mechanism for post-marketing surveillance, such as the UK Shunt Registry [11], should be established to closely monitor the proGAV performance *in-vivo*.

## Conclusion

Testing of the Miethke proGAV revealed it to be a reliable model that performed well *in vitro*. The brake system performed well and should minimise accidental resetting due to MRI scanning. The anti-siphon also works well and should help reduce posture-related over-drainage.

## Competing interests

The author(s) declare that they have no competing interests.

## Authors' contributions

All authors contributed equally to the study and they have read and approved the final manuscript.
